# How common is low pay in Britain? New findings from linked data

**DOI:** 10.23889/ijpds.v8i2.2221

**Published:** 2023-09-14

**Authors:** John Forth, Alex Bryson, Van Phan, Lucy Stokes, Felix Ritchie, Damian Whittard

**Affiliations:** 1 City, University of London, London United Kingdom; 2 University College London, London, United Kingdom; 3 University of the West of England, Bristol, United Kingdom; 4 National Institute of Economic and Social Research (NIESR), London, United Kingdom

## Objectives

The Annual Survey of Hours and Earnings (ASHE) is the main source of public statistics on low pay in Britain. As part of the ADR-funded Wage and Employment Dynamics Project, we identify and adjust for non-response biases in ASHE and generate new estimates of the incidence of low pay.

## Method

We linked the ASHE data to the Business Structure Database – a research-ready version of the UK’s official register of businesses. This linked dataset enabled us to identify which types of employers were more or less likely to respond to ASHE in a given year, and to generate non-response adjustments to the existing ASHE weights.

We then used the unique personal identifier on ASHE to link observations across years. We compared rates and correlates of longitudinal attrition in ASHE with rates and correlates of employment exit observed in the ONS Annual Population Survey, generating longitudinal weights to account for non-random attrition.

## Results

We find that jobs in smaller organisations, younger organisations and those in the private sector are under-represented in the annual achieved samples from ASHE, relative to their prevalence in the wider economy. The percentage of jobs paid at or below the National Minimum Wage is under-estimated by around one fifth if one does not take account of these cross-sectional response biases.

We find that longitudinal attrition is more likely to affect younger employees and those with low job tenure. However, we do not find that estimates of the rate at which employees move off the National Minimum Wage to higher rates of pay are biased by non-random patterns of longitudinal attrition.

## Conclusion

Data linking enables us to identify observable response biases in the UK’s official source of earnings statistics (ASHE). These biases affect our view of the bottom of the wage distribution, and have the potential to affect decisions around a key area of government labour market policy.

